# Impact of non-pharmaceutical interventions, weather, vaccination, and variants on COVID-19 transmission across departments in France

**DOI:** 10.1186/s12879-023-08106-1

**Published:** 2023-03-30

**Authors:** Juliette Paireau, Marie-Laure Charpignon, Sophie Larrieu, Clémentine Calba, Nathanaël Hozé, Pierre-Yves Boëlle, Rodolphe Thiebaut, Mélanie Prague, Simon Cauchemez

**Affiliations:** 1 Mathematical Modelling of Infectious Diseases Unit, Institut Pasteur, Université Paris Cité, CNRS UMR 2000, Paris, France; 2 Infectious Diseases Department, Santé Publique France, Saint Maurice, France; 3 Institute for Data, Systems, and Society (IDSS), Cambridge, MA USA; 4 Computational Health Informatics Program, Boston Children’s Hospital, Boston, MA USA; 5 University of Bordeaux, Inria Bordeaux Sud-Ouest, Inserm, Bordeaux Population Health Research Center, SISTM Team, UMR1219, Bordeaux, France; 6 Regions Department, Regional Office Nouvelle-Aquitaine, Santé publique France, Bordeaux, France; 7 Regions Department, Regional Office Provence-Alps-French Riviera and Corsica, Santé Publique France, Marseille, France; 8 INSERM, Sorbonne Université, Institut Pierre Louis d’Epidémiologie et de Santé Publique, Paris, France

**Keywords:** COVID-19, Non-pharmaceutical interventions, Vaccination, Weather, Reproduction number, Regression model

## Abstract

**Background:**

Multiple factors shape the temporal dynamics of the COVID-19 pandemic. Quantifying their relative contributions is key to guide future control strategies. Our objective was to disentangle the individual effects of non-pharmaceutical interventions (NPIs), weather, vaccination, and variants of concern (VOC) on local SARS-CoV-2 transmission.

**Methods:**

We developed a log-linear model for the weekly reproduction number (R) of hospital admissions in 92 French metropolitan departments. We leveraged (i) the homogeneity in data collection and NPI definitions across departments, (ii) the spatial heterogeneity in the timing of NPIs, and (iii) an extensive observation period (14 months) covering different weather conditions, VOC proportions, and vaccine coverage levels.

**Findings:**

Three lockdowns reduced R by 72.7% (95% CI 71.3–74.1), 70.4% (69.2–71.6) and 60.7% (56.4–64.5), respectively. Curfews implemented at 6/7 pm and 8/9 pm reduced R by 34.3% (27.9–40.2) and 18.9% (12.04–25.3), respectively. School closures reduced R by only 4.9% (2.0–7.8). We estimated that vaccination of the entire population would have reduced R by 71.7% (56.4–81.6), whereas the emergence of VOC (mainly Alpha during the study period) increased transmission by 44.6% (36.1–53.6) compared with the historical variant. Winter weather conditions (lower temperature and absolute humidity) increased R by 42.2% (37.3–47.3) compared to summer weather conditions. Additionally, we explored counterfactual scenarios (absence of VOC or vaccination) to assess their impact on hospital admissions.

**Interpretation:**

Our study demonstrates the strong effectiveness of NPIs and vaccination and quantifies the role of weather while adjusting for other confounders. It highlights the importance of retrospective evaluation of interventions to inform future decision-making.

**Supplementary Information:**

The online version contains supplementary material available at 10.1186/s12879-023-08106-1.

## Introduction

Since the beginning of the coronavirus disease 2019 (COVID-19) pandemic, several factors have contributed to the transmission dynamics of severe acute respiratory syndrome coronavirus 2 (SARS-CoV-2) in time and space. First, many countries around the world have implemented non-pharmaceutical interventions (NPIs), such as lockdowns, curfews, and school closures [1]. Before the introduction of vaccines, NPIs were the primary means to control disease spread. By the end of 2020 onwards, mass vaccination campaigns have helped mitigate the transmission of SARS-CoV-2 [2], while the concurrent emergence of more transmissible and immune escape variants of concern (VOC) has fostered virus spread [3]. Finally, the weather may also have modulated disease transmission [4]. Quantifying the relative contributions of each of these factors is key to better anticipate epidemic trends and guide future control strategies. However, this is challenging due to potential confounding, interaction effects, and a lack of identifiability of single effects when interventions or other factors are concomitant.

Several studies have investigated the effectiveness of NPIs at reducing SARS-CoV-2 transmission. The vast majority consisted of meta-analyses that combined data from multiple countries [1, 5–10]. However, conclusions drawn from such international comparisons may be affected by differences in local settings, data quality, NPI definitions, and population adherence to NPIs. In addition, most of these studies have only estimated the effect of NPIs during the first pandemic wave. Only a few have examined how the magnitude of NPI effects may have changed over time and in subsequent COVID-19 waves [11, 12]. In particular, the effect of NPIs that were only applied later in the pandemic, such as curfews, is still unclear. In addition to NPIs, the influence of weather on disease spread has been much debated [13, 14]. Previous statistical studies investigating the role of weather variables generally relied on single estimates of the reproduction number R, measured at different locations [4, 15–17] and early in the pandemic only. Thus, although these studies were timely and informative, they only covered a limited time period, when weather was likely less important to disease spread than governmental restrictions [18]. Importantly, only a limited number of meteorological studies controlled for other factors such as NPIs [16, 19, 20]; yet, not adjusting for sources of confounding may lead to spurious associations between weather and transmission. Now that data from a longer timespan are available, the role of weather conditions can be better elucidated.

To disentangle the effects of NPIs, weather, vaccination, and VOC on local SARS-CoV-2 transmission, we developed a statistical model to explain the time-varying reproduction number R reconstructed from the dynamics of hospital admissions, at the departmental level in metropolitan France, from March, 2020 to May, 2021. First, we leveraged the homogeneity in data collection and NPI definitions across departments. Indeed, in France, the number of patients hospitalized with COVID-19 was monitored through a single surveillance system implemented in all departments. In addition, most of the decisions on NPI implementation were made in a centralized manner. Such a standardized approach enabled harmonization of both data collection processes and NPI definition across departments, which benefited our study. Second, we leveraged spatial heterogeneity in the timing of NPI implementation. For example, lockdowns, curfews, and school closures were not systematically implemented at the same time in all departments, depending on the phase of the pandemic. This pattern in the timing of NPIs allowed us to circumvent the difficulty of assessing the impact of NPIs arising when measures are applied simultaneously across locations. Third, our study spanned a long observation period (14 months) that included varying weather conditions, VOC proportions, and vaccine coverage levels. This extensive study period covered three pandemic waves, thereby allowing us to examine the impact of successive NPIs, vaccine distribution, and the emergence of VOC. Capturing a full seasonal cycle allowed us to quantify the role of weather.

## Materials and methods

### COVID-19 data

Hospital data were obtained from the SI-VIC database, which is the national inpatient surveillance system used during the pandemic. This database is maintained by the ANS (Agence du Numérique en Santé) and provides real-time data on COVID-19 patients hospitalized in French public and private hospitals. All cases are either biologically confirmed or present with a PET scan image highly suggestive of SARS-CoV-2 infection. New daily hospital admissions were defined as the incremental number of patients admitted to a general ward or intensive care unit, indexed by date of admission (rather than date of reporting). Data were aggregated by department (administrative unit), based on hospital location. Of note, metropolitan France consists of 96 departments, with a median population size of 600,000 inhabitants.

### Covariates

We first selected covariates on the basis of their causal plausibility and gathered data on NPIs, VOC proportion, vaccine coverage, weather, mobility and demography.

To define covariates relative to NPIs, we collected data on the timeline of curfews, lockdowns, reopening periods following the lockdowns and during which restrictions were progressively lifted, as well as periods of more moderate restrictions (between any two lockdowns), using a combination of governmental websites, press articles, and Wikipedia pages. We also collected data on pandemic-related school closures (full or partial) and regular school holidays. Official dates were extracted from the Ministry of Education website (www.education.gouv.fr/calendrier-scolaire-100148). We measured an overall effect of school closures (whether pandemic-related or regular), with separate effects for summer and Christmas holidays.

The time-varying proportion of VOC was estimated using SIDEP (Système d’Information de Dépistage Populationnel—Information system for population-based testing) database, which is the national surveillance system describing RT-PCR and antigen test results arising from all private and public French laboratories. Test results are reported by date of nasopharyngeal swab and include patient information such as residential zip code. Aggregated data are publicly available (https://www.data.gouv.fr/fr/datasets/donnees-de-laboratoires-pour-le-depistage-indicateurs-sur-les-variants/). The proportion of VOC was assessed among positive RT-PCR or antigen test results, using RT-PCR screening kits. The three main VOC circulating during the study period were Alpha, Beta, and Gamma. Data on variants were available from February 15, 2021 onwards. Before this date, the proportion of VOC was imputed by fitting a separate logistic regression model for each department. We assumed absence of VOC before December 15, 2020 (Additional file 1: Fig. S1).

Vaccination data were obtained from the VAC-SI database, the national information system developed by the French Health Insurance to monitor the implementation of vaccination campaigns. Data are publicly available (www.data.gouv.fr/fr/datasets/donnees-relatives-aux-personnes-vaccinees-contre-la-covid-19-1/) and include both daily first-dose and full vaccine coverage time series, stratified by age group, and department, since the start of vaccine distribution in December 2020.

Weather data—including temperature, absolute humidity, and relative humidity– were obtained from Météo France/PREDICT Services for 112 weather stations nationally. We also included the IPTCC index *(Index PREDICT de transmissivité climatique de la COVID-19*) which characterizes weather conditions favoring SARS-CoV-2 transmission [21]. Data were averaged by department when necessary (82% of departments contain only one station).

Mobility data were obtained from Google mobility reports (www.google.com/covid19/mobility/). They describe the change in time spent at points of interest compared to a five-week baseline period (Jan 3–Feb 6, 2020). The six points of interest are: residential (time spent at home), workplaces, grocery and pharmacy, retail and recreation, parks, and transit stations. Data were available by department.

Finally, we included demographic data by department (population count and density), as obtained from the National Institute of Statistics and Economic Studies (https://www.insee.fr/fr/statistiques/4989753?sommaire=4989761).

### Statistical analyses

**Fig. 1:**
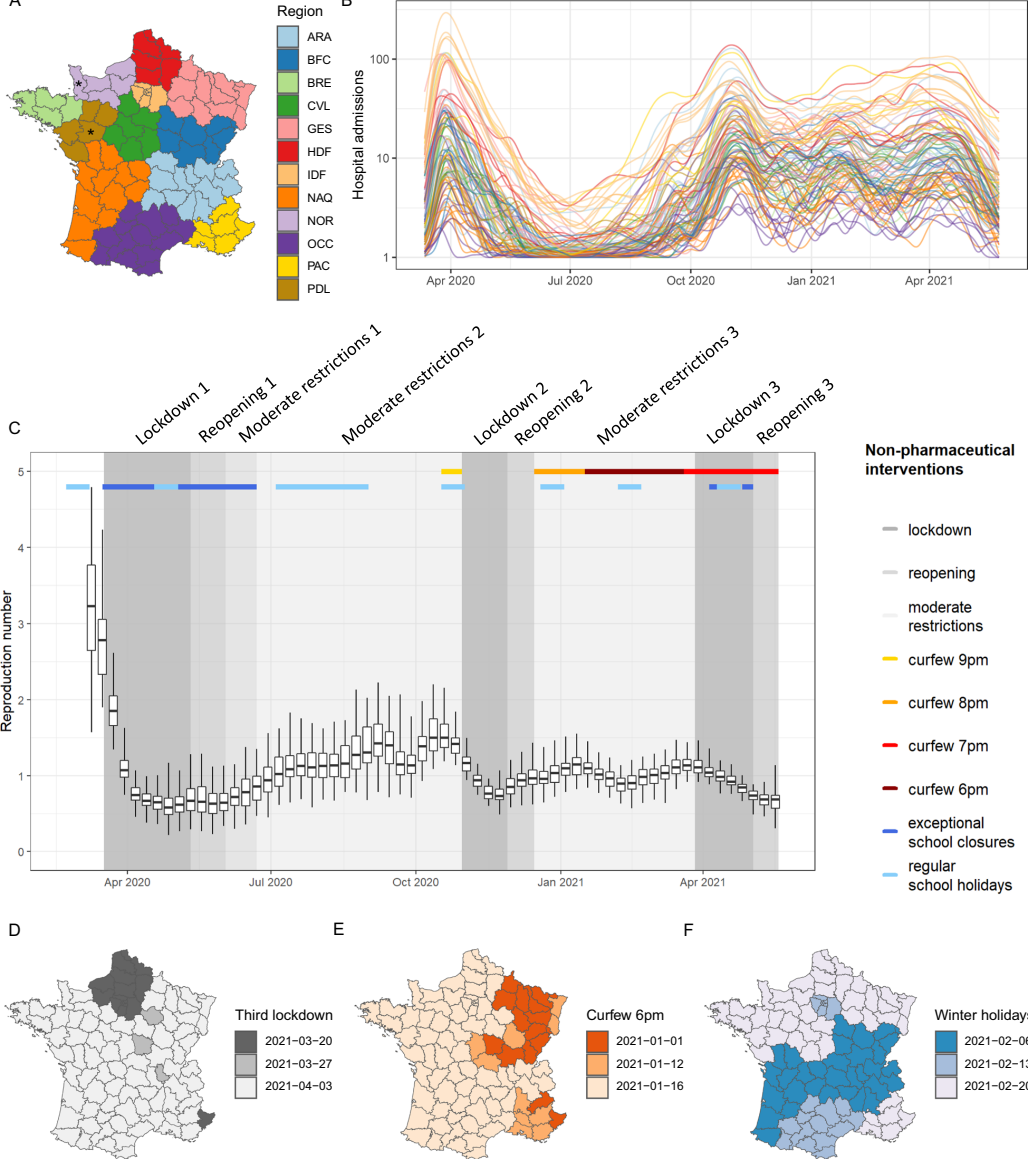
Dynamics of the COVID-19 epidemic and timing of non-pharmaceutical interventions across departments in metropolitan France, week 11–2020 to week 20–2021. **A** Map of departments, colored by region. The two departments marked by an asterisk were excluded, as well as Corsica (not shown), due to missing covariates. **B** Time series of new daily hospital admissions by department (logarithmic scale). **C** Temporal evolution of the reproduction number R by department and by week, overlaid with the timeline of non-pharmaceutical interventions. Boxplots feature the 2.5th, 25th, 50th, 75th, and 97.5th percentiles. Non-pharmaceutical interventions are shown taking the Rhône department as an example. **D** Timing of the third lockdown by department. **E** Timing of the 6 pm curfew by department. **F** Start dates of 2021 winter holidays by department

We analysed data collected from week 11–2020 (March 9–15, 2020) to week 20–2021 (May 17–23, 2021), in 92 of the 96 departments of metropolitan France (Fig. 1A). Four departments (Maine-et-Loire, Manche, Corse-du-Sud, and Haute-Corse) were excluded due to missing covariates. To remove random noise and weekend effects, daily hospital admission time series were smoothed using local polynomial regression (Fig. 1B). Using the package EpiEstim of the R software, we computed the reproduction number R on the smoothed series of each department, over seven-day rolling windows. The reproduction number is the average number of secondary cases caused by an infected individual. We used a gamma distribution with a mean of 7 days and a standard deviation of 5.2 days for the generation time [22].

We developed a log-linear mixed-effects model for the reproduction number $R_{ij}$ in department *i* in week *j*:

$$\log \left( {R_{ij} } \right) = \alpha + \beta_{k} \cdot X_{ijk} + \delta_{i} + \varepsilon_{ij}$$

where α is an intercept, $X_{ijk}$ are k covariates, $\beta_{k}$ are the associated regression coefficients (fixed effects), $\delta_{i}$ are department-level random effects following a Gaussian distribution, and $\varepsilon_{ij}$ is a Gaussian error term. We performed the analysis on a weekly scale to reduce temporal autocorrelation. Covariates with daily granularity were averaged by week. Weather covariates were introduced into the model either linearly or as cubic B-splines. Splines with different degrees of freedom were compared using the Akaike Information Criterion (AIC) and the model that yielded the lowest AIC was selected. The model was fitted by maximum likelihood using the R package nlme. Confidence intervals (CI) for the parameters were obtained using a normal approximation to the distribution of the maximum likelihood estimators. We also tested a model with spatially-correlated department-level random effects, using the R-INLA package to assess spatial autocorrelation.

To account for the delayed effects of covariates on hospital admission dynamics, we applied an 11-day lag (5 days for the incubation period and 6 days for the delay between symptom onset and hospital admission [22]) for NPIs, proportion of the population infected, weather conditions, and mobility. For first-dose vaccination, we applied an 18-day lag (12 days for the build-up of immunity [23] and 6 days for the delay between symptom onset and hospital admission). For full vaccination, we reduced this delay to 13 days [23]. For VOC proportion (based on testing data), we applied a three-day lag (mean delay between test and hospital admission observed in French data). In a sensitivity analysis, we tested for additional lags (± 2 days) and selected the value leading to the lowest AIC. The lags were applied to daily data, before covariates were averaged by week.

We first built a baseline model that only included lockdowns, reopenings and moderate restrictions. Then, we incorporated additional covariates using a forward selection procedure. At each step, the covariate leading to the lowest AIC was introduced in the model, until no additional covariate improved the AIC. Using the AIC allows balancing goodness-of-fit and model complexity, by penalizing models with a large number of parameters, in order to reduce overfitting and instead favor parsimony.

In order to assess the impact of smoothing on the robustness of our estimates, we also performed a sensitivity analysis by running the model on the raw (non-smoothed) hospitalization data.

To further characterize the individual effects of key covariates on transmission and hospital admissions, we determined the expected R for an average department under two distinct counterfactual scenarios: (i) without the effect of vaccination, and (ii) without the effect of VOC. We then projected the expected number of new hospital admissions at the national level under such scenarios, from January 11, 2021 onwards.

## Results

### COVID-19 dynamics, NPIs and holidays in France

The median reproduction number R was above 2.5 during the first two weeks and oscillated between 0.6 and 1.9 during the rest of the period (Fig. 1C). Three national lockdowns were implemented. The first started on March 17, 2020 and lasted approximately two months. The second and third lockdowns were initiated on October 30, 2020 and April 3, 2021, respectively, and lasted one month each (Fig. 1C). The third lockdown started one or two weeks earlier in 19 departments than in the rest of the country (Fig. 1D). During the first lockdown, local movements were restricted to a maximum of 1 km around the place of residence for no more than one hour, gatherings in public space were forbidden, and non-essential shops, parks, bars, and restaurants were closed (Additional file 1: Table S1). During the second and third lockdowns, similar measures were imposed, but gatherings of up to 6 people were allowed in public space and parks remained open. During the third lockdown, local movements around the place of residence were allowed up to 10 km. Each lockdown was followed by a reopening phase, during which some (but not all) of the restrictions were lifted. For instance, during the reopening period that followed the first lockdown, local movements were allowed and non-essential shops were open, but inter-regional movements were limited to 100 km around the place of residence and bars and restaurants remained closed. Between lockdowns, several restrictions were applied, including public events limited to 5000 persons and partial closing of cultural places (Additional file 1: Table S1). An overnight curfew starting at 9 pm was first implemented in 16 departments (9 metropolises) on October 17, 2020, followed by 38 other departments on October 24, 2020. At the end of the reopening period that followed the second lockdown, on December 15, 2020, a curfew starting at 8 pm was implemented in all departments. This curfew was then moved to an earlier start at 6 pm. The measure was first applied in 15 departments on January 2, 2021 and in 10 more departments on January 12, 2021, before extension to the whole country on January 16, 2021 (Fig. 1E). On March 20, 2021, the national curfew was pushed to 7 pm. There were five regular holiday periods: 2 weeks in February/March, 2 weeks in April, 2 months in July–August, 2 weeks in November and 2 weeks in December/January (“Christmas holidays”). The timing of holiday periods may vary by department (Fig. 1F). In addition to regular school holidays, schools remained fully or partially closed during the first lockdown through June 22, 2020, and during the third lockdown (Fig. 1C). However, they remained open during the second lockdown.

### Multivariable model

**Fig. 2:**
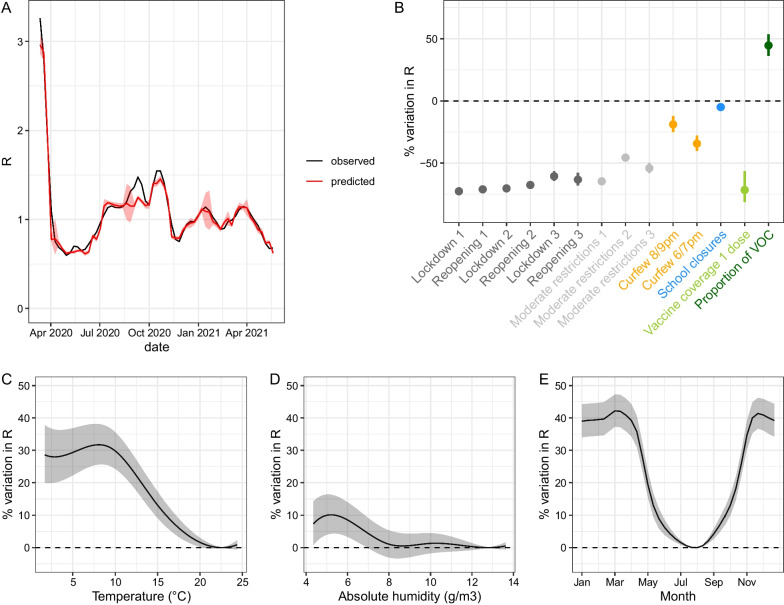
Goodness-of-fit and estimated effects of covariates included in the multivariable model on the reproduction number R (in percentage of variation). **A** Trajectory of R estimated by the full model for an average department (red) compared to mean R (black). **B** Effects of linear and categorical covariates. Of note, the effect shown for first-dose vaccine coverage and the proportion of VOC corresponds to a covariate value of 100% (i.e., reflecting a fully vaccinated population and maximum prevalence of variants). **C** Non-linear effect of temperature. **D** Non-linear effect of absolute humidity. **E** Estimated seasonality of COVID-19 based on average temperature and absolute humidity observed over 1981–2010 in metropolitan France, after adjusting for other covariates. For **C**, **D** and **E**, fitted lines and their 95% confidence intervals show the estimated percentage of variation in R with respect to a baseline set to the value at the trough of the corresponding curve for weather variables. For **C** and **D**, the range of the x-axis is determined by the 2.5–97.5th percentile of the weather variable distribution

In addition to lockdowns, reopenings, and moderate restrictions, the final multivariable model included curfews, school closures, first-dose vaccine coverage, proportion of VOC, temperature, and absolute humidity (Additional file 1: Fig. S2). The lowest AIC was obtained for the following lags: 11 days for NPIs and weather variables, 20 days for vaccine coverage, and 5 days for the proportion of VOC. The correlation between the observed and fitted values of R was relatively high, although the fitted values presented lower variability than the observed values: the proportion of the variance explained by the full model, including fixed and random effects, reached 63.8%, as estimated based on the conditional R^2^ [24] (Additional file 1: Figure S3). Except for the small peak observed in September 2020, the average trajectory of R was well captured by the model (Fig. 2A).

School closures, excluding summer and Christmas holidays, reduced R by 4.9% (95% CI 2.0–7.8) (Fig. 2B). Covariates characterizing summer and Christmas holidays were not statistically significant and therefore not included in the final model. The earlier overnight curfews started, the stronger their effect on transmission: R was reduced by 18.9% (12.0–25.3) for the 8/9 pm curfews and by 34.3% (27.9–40.2) for the 6/7 pm curfews. The first lockdown reduced R by 72.7% (71.3–74.1). Combined with school closures, it yielded a reduction in R of 74.1% during the corresponding time period (non-linear effect). The second lockdown reduced R by 70.4% (69.2–71.6), with schools remaining open. The third lockdown reduced R by 60.7% (56.4–64.5). Combined with school closures and the nightly curfew starting at 7 pm, it yielded a reduction in R of 75.4%. Reductions in R observed during reopening periods following lockdowns were similar to those measured during lockdowns. The reduction in transmission associated with moderate restrictions ranged from 45.6% to 64.7%, depending on the time period. Furthermore, we estimate that 100% first-dose vaccine coverage would have reduced R by 71.7% (56.4–81.6). In practice, this effect induced a 17.6% and 34.1% reduction in transmission in the departments with the lowest and highest first-dose vaccine coverage at the end of the observation period, respectively. In contrast, a 100% proportion of VOC (mainly Alpha) increased transmission by 44.6% (36.1–53.6) compared with the period during which the historical strain was predominant. Finally, among the weather conditions that we considered, temperature was the factor that improved the model the most, followed by absolute humidity. The AIC was lower when these covariates were included as splines (degrees of freedom of 5 and 6, respectively) rather than as linear effects. We found that R was the lowest at 22.6 °C and the highest at 8.1 °C. Between the minimum and the maximum values, it increased by up to 31.7% (25.6–38.1) (Fig. 2C). With respect to absolute humidity, R was the lowest at 12.8 g/m^3^ and increased by up to 10.1% (4.3–16.3) to reach a maximum at 5.2 g/m^3^ (Fig. 2D). Considering a national average of weather conditions, we predicted that the transmission rate was the highest in November-March and the lowest in July–August, with an overall amplitude of 42.2% (37.3–47.3) (Fig. 2E). Estimated department-level random effects were small, ranging between − 1.10^–8^ and 1.10^–8^ (Additional file 1: Fig. S4). We found no difference when including spatially-correlated random effects and thus opted for the simpler model using Gaussian random effects. A model without random effects only had a slightly higher AIC (difference of 2 points).

### Sensitivity analyses

When running the model on R estimated from non-smoothed hospitalization data, we found very close central estimates, with slightly wider confidence intervals (Additional file 1: Fig. S5). The school effect was no longer statistically significant. The effects of weather variables were up to 5% higher but not significantly different from the main analysis.

When adding summer and Christmas holidays in the final multivariable model, the results were not modified, suggesting no additional confounding effect owing to these two covariates (Additional file 1: Fig. S6).

### Counterfactual scenarios

**Fig. 3:**
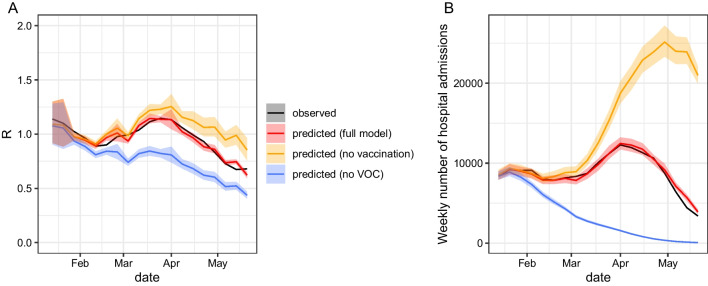
Projected trajectory of R and hospital admissions from January 11, 2021, under different scenarios: the full model, a model without vaccination and a model without VOC. **A** Mean trajectory of R. **B** Number of national-level weekly hospital admissions (summed across all departments)

In a first counterfactual scenario, we showed that, for the set of NPIs that were implemented at the time, R would have remained above 1 for three additional weeks (until week 17 vs week 14 in reality) in the absence of vaccination (Fig. 3A). Such a scenario would have resulted in a peak of 25,000 new weekly hospital admissions in May 2021 (Fig. 3B), higher than observed during the first wave in March 2020.

In a second counterfactual scenario, we showed that, in the absence of VOC, the epidemic could have been contained earlier, with R remaining below 1 in February–May2021 (Fig. 3A) and the observed increase in hospital admissions (third wave for France) would not have occurred (Fig. 3B).

## Discussion

The methodology used in this study allowed us to disentangle the effects of multiple factors on the reproduction number across French departments, over an extensive observation period spanning three distinct pandemic waves. In particular, our multivariable model demonstrated the strong beneficial effect of NPIs and vaccination on COVID-19 transmission. It also highlighted the detrimental role of emerging variants. Importantly, it enabled quantifying the impact of weather conditions on local transmission while adjusting for other covariates.

The final mixed-effects model presented in this study was able to replicate the temporal dynamics of the reproduction number observed in metropolitan France during the first 14 months of the COVID-19 pandemic. The model closely matched the data but for a short period in September 2020, where the temporary increase in the reproduction number was not captured. This sporadic increase coincided with the end of the summer holidays and a period when the avoidance of social gatherings fell to a low level [25]. Yet information on such individual behaviours at the department level was not available for inclusion in the regression model. However, both the return-to-school and the resurgence of social gatherings presumably contributed to shape the change in transmission, in addition to weather conditions and decisions made by health authorities. In addition, the model did not fully capture the high variability observed in the department-level reproduction numbers at the beginning of the study period (March 2020). Such heterogeneity in the early spread of the epidemic might be due to other factors that we did not account for: for example, it has been shown that, before the first lockdown, R was larger in regions where the virus was first introduced, and their neighboring regions [4].

The multivariable model yielded a gradient in the effectiveness of lockdown measures. The first lockdown had the largest impact, followed by the second and the third, which is consistent with international data [11]. This could be explained by the more restrictive measures implemented during the first lockdown, but also potentially by increasing pandemic fatigue, which may have resulted in mobility rebounds, more frequent social interactions, and decreasing compliance with preventive measures [26]. Of note, we considered that the effect of each lockdown was constant over the duration of the intervention, although it might vary over time due to behavioral patterns [7, 26]. Thus, our estimates of the impact of lockdowns on the reproduction number must be interpreted as averaged effects. We found that transmission during the reopening phases following the lifting of lockdowns remained similar to that observed during lockdowns. One possible interpretation is that population behavior did not change immediately with policies: the population may have continued to adhere to public health measures, such as physical distancing during the reopening phase, e.g., due to fear of a COVID-19 rebound and preventive habits taken during lockdowns. Early-pandemic association studies spanning over 131 countries also reported that more time was needed to observe the effects of relaxing NPIs than to detect those resulting from the introduction of new restrictions [7]. Moreover, during reopening phases, restrictions were only partially lifted and remained quite intense (Additional file 1: Table S1). Even during intermediate periods of moderate restrictions, the reduction in R was substantial, ranging from 45.6 to 64.7%. This likely reflects the additional contributions of other NPIs such as mask-wearing, hygiene measures, contact tracing and case isolation. Our framework did not allow evaluation of individual effects of such NPIs, which were applied throughout the study period. Moreover, due to collinearity, the effects of specific policies described in Additional file 1: Table S1 (e.g. shop closures, restaurant closures etc.) could not be evaluated separately and were therefore collapsed into broader categories (lockdowns, reopenings and moderate restrictions). Interestingly, overnight curfews considerably reduced SARS-CoV-2 transmission, corroborating results obtained in French Guiana (up to 35% reduction in transmission rates) [27] and in Quebec, Canada (similar reductions in human mobility) [28]. Moreover, we found that curfews starting earlier in the evening (6/7 pm) had a larger impact on transmission than curfews starting later (8/9 pm).

Perhaps more surprisingly, school closures were found to have only a limited effect on transmission. Importantly, school closures did not uniformly affect all households and instead led to disparities in childcare across families that may potentially hinder their effect. As highlighted in other studies, policy decisions about school closures or hybrid school schedules often need to be weighted against the risks of disease transmission to elderly populations associated with increased intergenerational contact rates that exacerbate their vulnerability due to weaker immune systems [29]. Our result differs from that of Nader et al. [1], who found that school closures were one of the most important NPIs in the 60 days following their implementation. In the French context, we believe that the enforcement of mask-wearing and barrier gestures at school was also an impactful NPI that might explain such a difference in magnitude: because these restrictions were applied concurrently, the effect of school closures may have been partially occulted. This observation is consistent with simulation-based scenarios tested in Saudi Arabia [30], where mask-wearing and physical distancing applied in schools were able to drastically reduce the effect of in-person education on SARS-CoV-2 transmission.

The role of weather conditions in COVID-19 transmission has been debated in the literature [15]. Here, after controlling for other confounding factors such as NPIs, we found a substantial effect of temperature (up to 31.7% variation in R), followed by absolute humidity (up to 10.1% variation), which led to a 42.2% variation in R between summer and winter months in France. Although both temperature and humidity were associated with SARS-CoV-2 transmission, the overall goodness-of-fit was found to be lower when using the compound IPTCC index. In the future, a different parametrization of this index may yield better performance. The estimate of the joint effect of temperature and absolute humidity was similar in magnitude and range to individual contributions reported by prior observational [17, 31, 32], in-vitro [33], and physio-mechanical studies [34]. Similar to Sera et al. [16], we found a non-linear relationship between weather variables and R. However, in our study, the peak of transmission was identified at slightly lower temperature and lower absolute humidity values. Pursuing such research in larger countries with more spatial heterogeneities in climatic conditions would be particularly valuable. Interestingly, we found that the temporal seasonality of SARS-CoV-2 transmission was similar to the known seasonality of influenza epidemics in temperate climates [35].

We found that variants of concern (mainly Alpha) increased the reproduction number by 44.6% (36.1–53.6). This is consistent with the effect of Alpha variant on transmission reported in the literature, ranging from about 25% to more than 90% [3, 36, 37]. Notably, the strength of our study is that it estimates the effect of VOC while simultaneously adjusting for weather conditions. Considering that VOC appeared in winter 2021 period and that their proportion substantially increased in February–March 2021, when temperatures were still low and favoured SARS-CoV-2 transmission, such an adjustment was deemed necessary. Our counterfactual scenario analysis showed that, in the absence of VOC, vaccination associated with moderate restrictions and curfew would have been sufficient to contain the historical virus. In the other counterfactual scenario, we showed that, without vaccination, the spread of VOC would have resulted in a peak of hospital admissions higher than observed during the first wave. However, it should be noted that these scenarios were not based on a dynamical transmission model. For instance, we did not account for the fact that if herd immunity was reached in the scenario without vaccination, the number of hospital admissions at the peak would have been lower than predicted by our model. In addition, in practice, it is likely additional measures would have been implemented that would have limited the impact on healthcare.

Although the multivariable model successfully captured the overall temporal dynamics of R, unexplained variability across departments remained. Spatial variation in the reproduction number may arise from underlying socio-demographic determinants such as age distribution, degree of urbanicity, or job market structure [38], which differ between departments. Therefore, we accounted for geographical variation through a department-level random effect on the reproduction number. However, the estimated magnitude of this effect was extremely small. Further, we did not find any statistically significant effect when additionally testing for a potential contribution of population density or population count, suggesting limited impact of population structure. Apart from spatial variation in the average reproduction number, small deviations from the average effect of a given intervention may exist among geographical units, due to spatial variations in determinants of population adherence to preventative health measures. Yet introducing the possibility of such variation brings challenges in parameter identifiability. As a result, model sparsity was preferred and department-specific effects on explanatory variables were not considered.

In this study, we used a regression analysis to quantify the impact of categorical (NPIs) and continuous (weather, vaccination…) covariates on a dependent variable (the reproduction number), a widely-accepted method in epidemiological modelling [7, 8, 15, 16]. Alternative approaches can be employed, such as event studies or difference-in-difference analyses [9]. Herein, their implementation is challenged by the multiplicity of interventions, sometimes deployed at different dates among departments. Importantly, our results are based on a retrospective observational study in which interactions, collinearity and mediation effects may occur. Therefore, the effects estimated here only reflect statistical associations and do not necessarily imply causal mechanisms. In addition, given the differential timeline of interventions, interpretation of their absolute effect should only be made within a specific context of implementation and cannot be directly extrapolated to other settings. Of note, we did not extend the study period further for two main reasons. First, no NPIs were implemented in France after May 2021. Second, the dynamics of the epidemic became mainly driven by the evolution of immunity in the population; capturing such effects would require a different modelling approach. In particular, the study should account for waning vaccine efficacy if extended to a longer time period. However, this adjustment was not deemed necessary here, given that most vaccinations only occurred at the end of the study period (Additional file 1: Fig. S2).

Our study has other limitations. First, our analysis relies on a two-step approach that omits uncertainty in the estimation of the reproduction number when evaluating the effect of NPIs and other factors. This may lead to an underestimation of the width of the confidence interval around the point estimate characterizing the effect of each factor. Second, the reproduction number was estimated using a constant generation time across the study period. However, the Alpha variant could have a slightly shorter generation time than the ancestral strain [39]. In that case, we would expect that our estimates of R at the end of the study period (when Alpha replaced the ancestral strain) were overestimated when R > 1 and underestimated when R < 1 [40]. This could have led to an overestimation of the effect size associated with interventions occurring at the end of the study period (e.g. the third lockdown). Third, R was estimated from hospital admissions and covariates were lagged to account for the delay between each covariate and hospital admission. This allowed us to test different lag values depending on the covariate but might not be accurate when the delay is long [40]. Other methods such as deconvolution can be used, provided that the delay distribution is well known; however, this method is sensitive to misspecification of the mean, variance, or form of the delay distribution [40]. Fourth, we could not exclude hospital-acquired infections from the times series of hospitalized patients due to a limitation of the dataset. This could have led to biased estimates of R if the proportion of hospital-acquired infections was not stable through time. However, most of SARS-CoV-2 transmission occurred in the community and hospital-acquired transmission only represented a small proportion of transmission events [41]; therefore, our results should not be substantially affected by the aggregation of settings (i.e. hospital and community). Similarly, we were not able to exclude hospitalizations of nursing home residents due to the limited granularity of the dataset. However, these represented only 5% of total hospitalizations in France over the study period; thus, including them should have a limited impact on our estimates of R.

In summary, through a multivariable analysis across 92 French departments, this study allowed disentangling the individual contribution of NPIs, weather, first-dose vaccination, and VOC proportion on local SARS-CoV-2 transmission during three successive pandemic waves. Our findings highlight the importance of retrospective evaluation of past interventions to inform future decision-making for better epidemic control.

## Implications of all available evidence

This study helps to better understand the underlying factors of COVID-19 dynamics observed during the first three waves of the pandemic in France. This retrospective evaluation of past interventions provides evidence base for informing future decision-making during epidemics. Our findings also highlight the need to account for the effect of weather conditions in SARS-CoV-2 transmission models.

## Supplementary Information

**Additional file 1: Table S1.** Description of the main non-pharmaceutical interventions (NPIs) applied during each lockdown, reopening, and period of moderate restrictions. **Figure S1.** Imputation of the proportion of VOC using a logistic regression. **Figure S2.** Time series of continuous covariates included in the final multivariable model. **Figure S3.** Calibration performance: correlation of observed R vs fitted R. **Figure S4.** Map (A) and histogram (B) of department-level random effects. **Figure S5.** Results of the sensitivity analysis using raw hospitalization data instead of smoothed data. **Figure S6.** Results of the sensitivity analysis including summer and Christmas holidays.

## Data Availability

The datasets generated and/or analysed during the current study are available in Gitlab (https://gitlab.pasteur.fr/mmmi-pasteur/covid19-departmental-model).
