# Supplementary material for: Impact of non-pharmaceutical interventions, weather, vaccination, and variants on COVID-19 transmission across departments in France
Source: BMC Infect Dis. 2023 Mar 30;23:190. doi: 10.1186/s12879-023-08106-1 (PMC10061408; doi:10.1186/s12879-023-08106-1)
Supplement: Supplementary file 1 — Additional file 1: Table S1. Description of the main non-pharmaceutical interventions (NPIs) applied during each lockdown, reopening, and period of moderate restrictions. Figure S1. Imputation of the proportion of VOC using a logistic regression. Figure S2. Time series of continuous covariates included in the final multivariable model. Figure S3. Calibration performance: correlation of observed R vs fitted R. Figure S4. Map (A) and histogram (B) of department-level random effects. Figure S5. Results of the sensitivity analysis using raw hospitalization data instead of smoothed data. Figure S6. Results of the sensitivity analysis including summer and Christmas holidays. [file 12879_2023_8106_MOESM1_ESM.docx]

**Additional file 1**

**Impact of non-pharmaceutical interventions, weather, vaccination, and variants on COVID-19 transmission across departments in France**

Juliette Paireau, Marie-Laure Charpignon, Sophie Larrieu, Clémentine Calba, Nathanaël Hozé, Pierre-Yves Boëlle, Rodolphe Thiebaut, Mélanie Prague, Simon Cauchemez


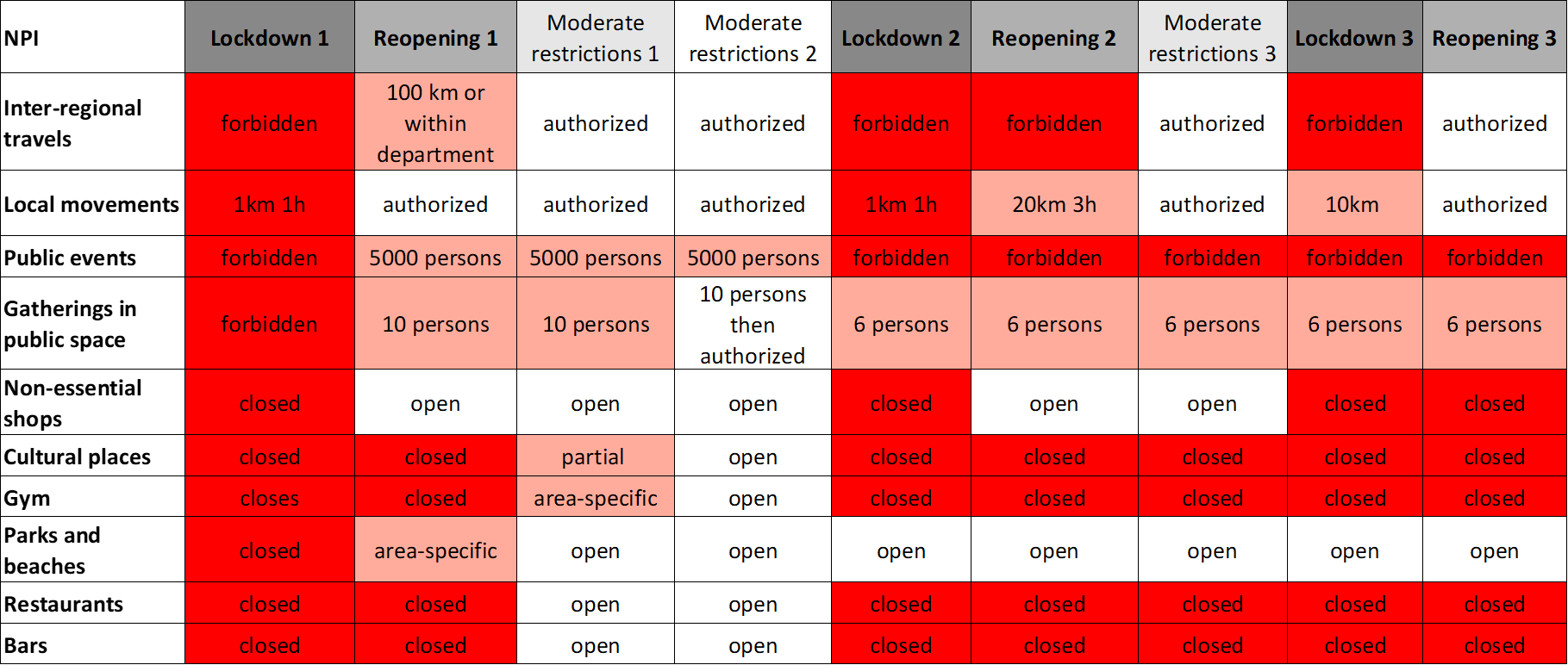


**Table S1: Description of the main non-pharmaceutical interventions (NPIs) applied during each lockdown, reopening, and period of moderate restrictions.** The darker the color, the more restrictive the measure.


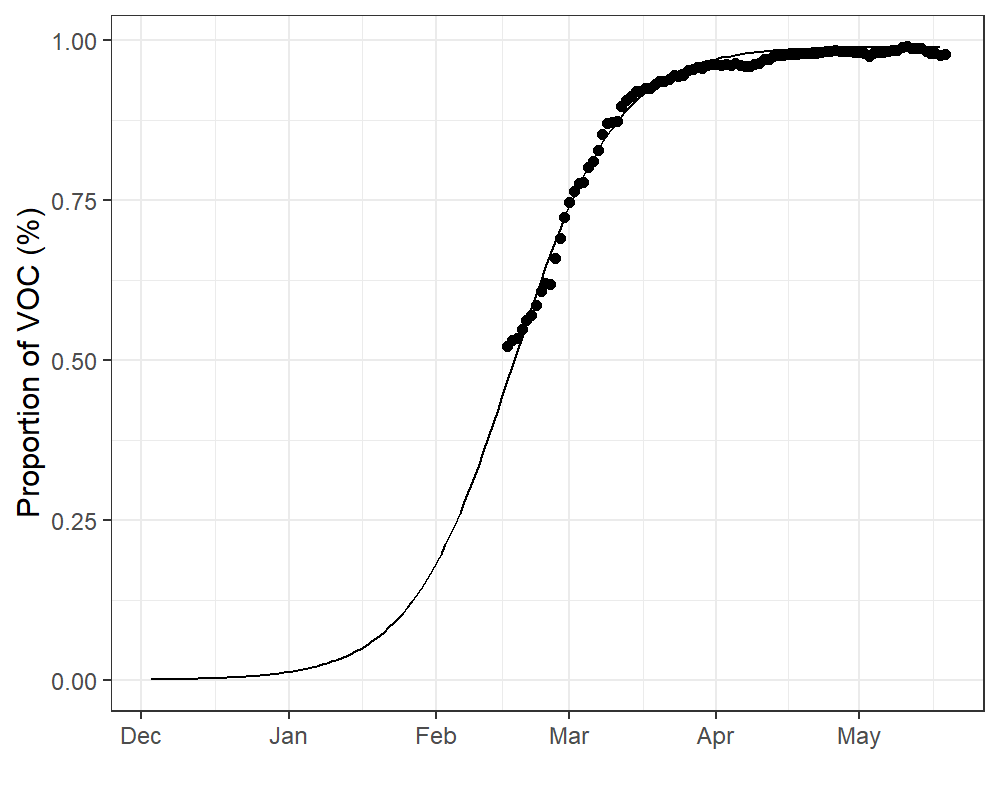


**Figure S1: Imputation of the proportion of VOC using a logistic regression.** The points represent available data and the line represents the logistic fit, taking the Rhône department as an example from December 2020 to May 2021.


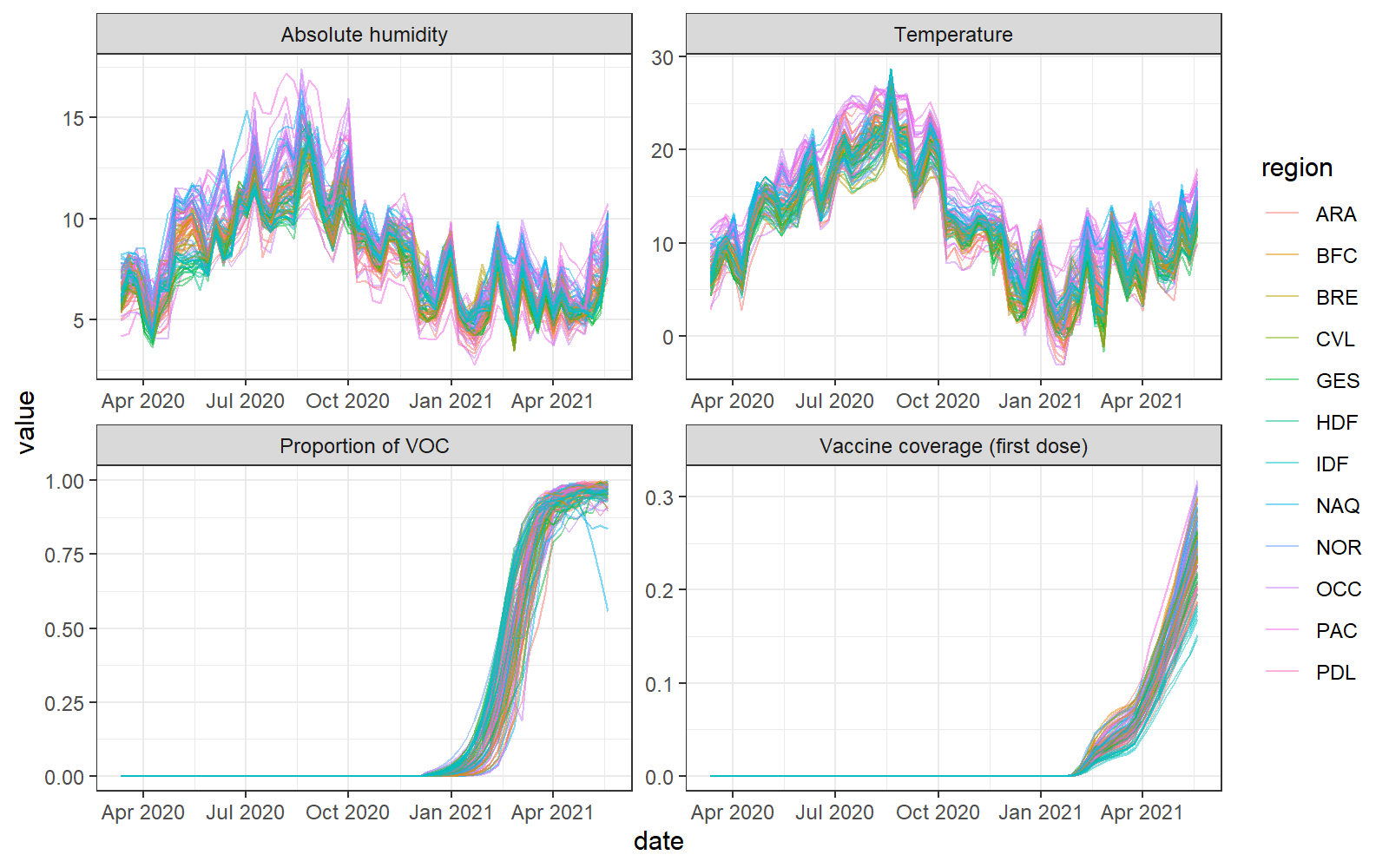


**Figure S2: Time series of continuous covariates included in the final multivariable model.** Each line represents a department and is colored by region. Regions: Auvergne-Rhône-Alpes (ARA), Bourgogne-Franche-Comte (BFC), Bretagne (BRE), Centre-Val de Loire (CVL), Grand Est (GES), Hauts-de-France (HDF), Île-de-France (IDF), Nouvelle-Aquitaine (NAQ), Normandie (NOR), Occitanie (OCC), Provence-Alpes-Côte d’Azur (PAC), and Pays de la Loire (PDL).


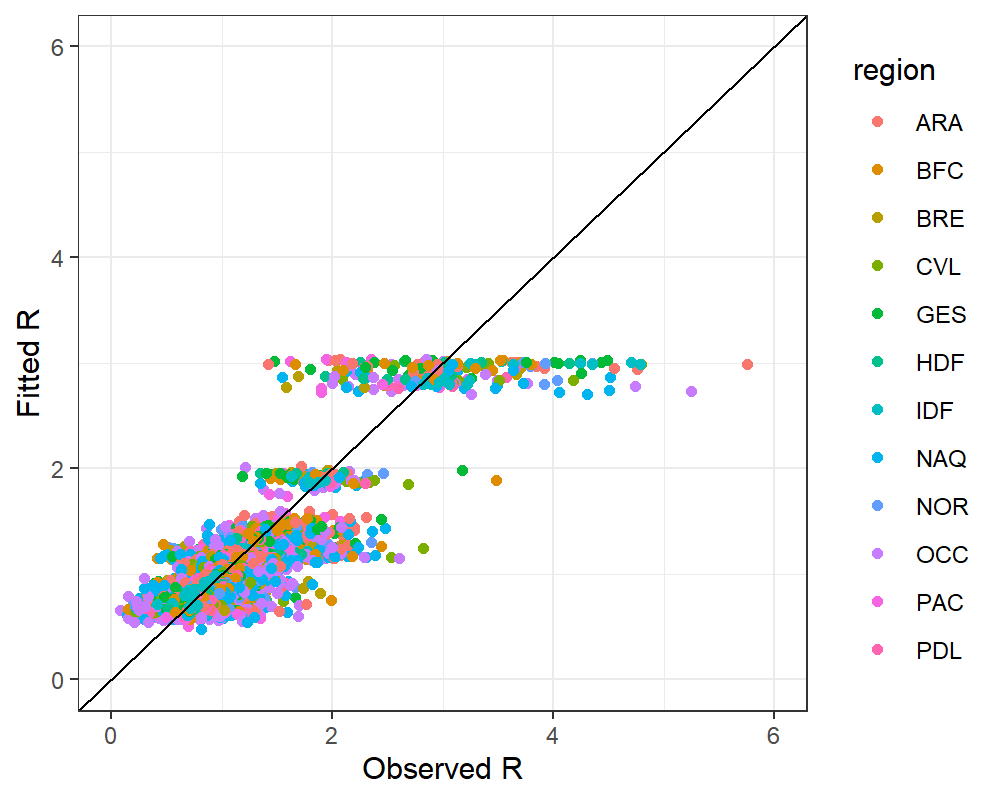


**Figure S3: Calibration performance: correlation of observed R vs fitted R.** Each point represents a department-week pair and is colored by region.

A B


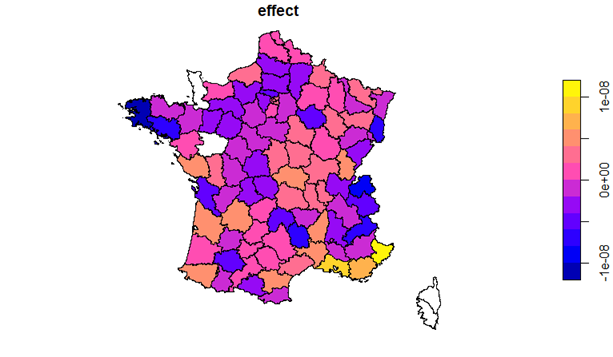

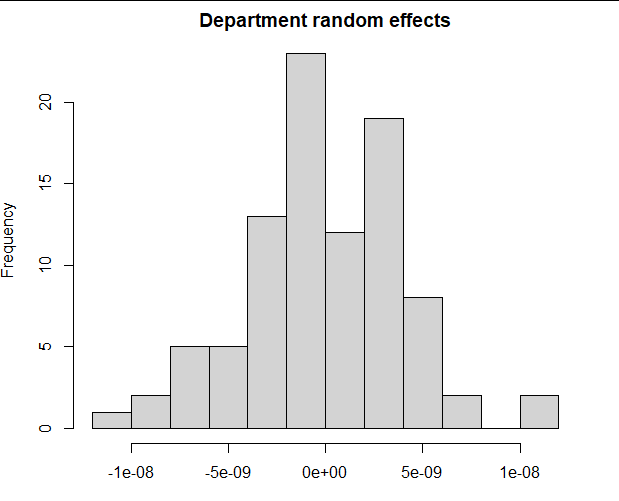


**Figure S4: Map (A) and histogram (B) of department-level random effects.**

**
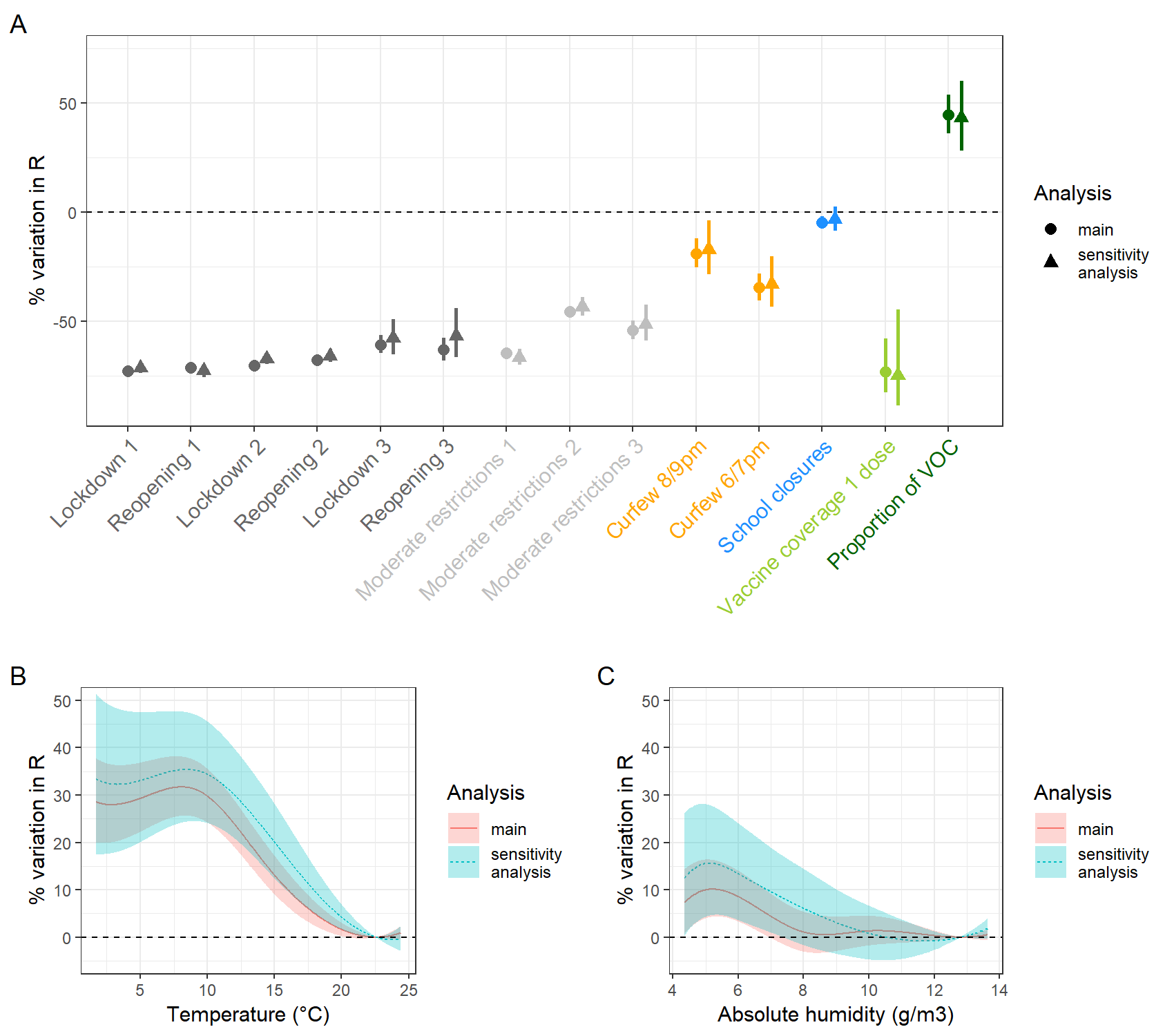
Figure S5: Results of the sensitivity analysis using raw hospitalization data instead of smoothed data.** (A) Effects of linear and categorical covariates included in the multivariable model on the reproduction number (in percentage of variation). (B) Non-linear effect of temperature. (C) Non-linear effect of absolute humidity.

**
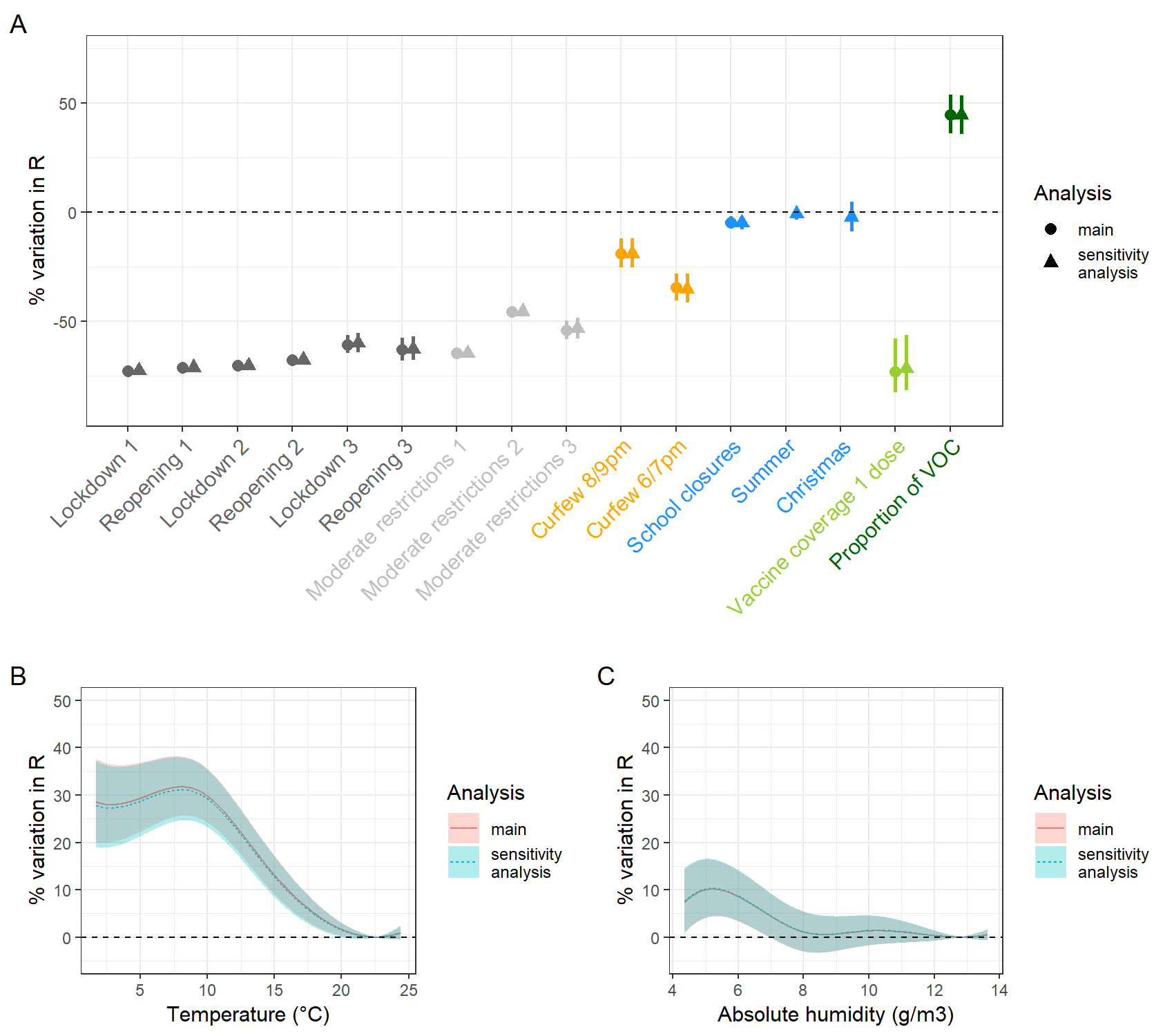
**

**Figure S6: Results of the sensitivity analysis including summer and Christmas holidays.** (A) Effects of linear and categorical covariates included in the multivariable model on the reproduction number (in percentage of variation). (B) Non-linear effect of temperature. (C) Non-linear effect of absolute humidity.
